# Comparing community-based monitoring to hospital-based care of patients with quiescent age-related macular degeneration: a qualitative study of patient and practitioner perspectives on acceptability and access

**DOI:** 10.1136/bmjopen-2025-101379

**Published:** 2026-02-04

**Authors:** Sofia Vougioukalou, Simon M Read, Judit Katalin Csontos, Aled Jones, Alijazy Jaber, Anitta Sharma, Konstantinos Balaskas

**Affiliations:** 1 Centre for Adult Social Care Research, Centre for Trials Research, Cardiff University, Cardiff, UK; 2 Adults and Communities Department, Torfaen County Borough Council, Pontypool, UK; 3 School of Healthcare Sciences, Cardiff University, Cardiff, UK; 4 Plymouth University, Plymouth, UK; 5 Moorfields Eye Hospital NHS Foundation Trust, London, England; 6 Institute of Ophthalmology, UCL, London, UK

**Keywords:** OPHTHALMOLOGY, GERIATRIC MEDICINE, Clinical Trial, SOCIAL MEDICINE, QUALITATIVE RESEARCH

## Abstract

**Objectives:**

This process evaluation explores patient and healthcare professional acceptability of community-based monitoring versus hospital-based care for patients with quiescent neovascular age-related macular degeneration (QnAMD).

**Design:**

Qualitative process evaluation was conducted as part of a randomised controlled trial.

**Setting:**

Six hospitals and six community-based practices.

**Participants:**

25 patients and 16 healthcare professionals (ophthalmologists and optometrists). This approach helped differentiate between common issues and those specific to community-based monitoring.

**Intervention:**

The Quality-Assured Follow-Up of QnAMD by non-medical practitioners trial aimed to examine whether non-medical practitioners follow-up patients with QnAMD in the community in a safe and clinically and cost-effective way. The process evaluation aimed to examine whether the intervention was acceptable by patients and professionals. The process evaluation was based on interviews which contained open-ended questions focused on patient experience and confidence in community-based care, issues concerning the practicalities of the organisation and management of the clinic, and resources including IT and digital equipment. The theory of acceptability framework was used to interpret the findings.

**Results:**

Patients reported positively on the experience of receiving QnAMD services in the community and highlighted staff professionalism and clear communication. Key themes were the proximity of care provision for patients, IT interoperability and the real-world costs of running the service. Some patients randomised to the hospital showed preference for the intervention to take place in the hospital, mediated mainly by prior experience of hospital care and travel distance. The location of the clinic and transport routes affected the experience of attending appointments, with strong preference expressed for proximity to one’s home. Inaccessibility due to non-modifiable internal building structures in the community and parking in hospital eye services was reported by a small proportion of patients. Healthcare professionals reported positively about their ability to deliver QnAMD services in community settings but raised concerns about the compatibility of technological infrastructure that facilitates the sharing of optical coherence tomography image and video files. Some optometrists were also concerned about the financial sustainability of the intervention after the end of the trial due to the costs involved in the administration of QnAMD follow-up care.

**Conclusions:**

The delivery of QnAMD services in the community by non-medical personnel was broadly accepted by both patients and practitioners. This implies that non-medical practitioners can follow up patients with QnAMD in the community in a safe way. Further research would be needed to establish whether similar results would be obtained during routine practice outside a research project and whether the long-term follow-up for QnAMD would be financially sustainable for independent as well as chain community optometry practices.

**Trial registration number:**

NCT03893474.

**Strengths and limitations of this study**

- To our knowledge, this is the first process evaluation of a randomised controlled trial of community-based eye care in the UK.
- The locations of the intervention were geographically spread across the UK with variation in sampling ensuring that patients randomised in the community as well as the hospital and professionals, and from diverse backgrounds, shared their views.
- Data saturation was reached within a sample of 25 patients and 16 professionals, indicating clear patterns of acceptability across the sample.
- The trial was suspended during COVID-19 for 102 days and this disrupted recruitment to the process evaluation, as well as opportunities for face-to-face interviews and observations.

## Introduction

 Interest in measuring and improving the appropriateness of eye care delivery in community settings is growing, with a particular focus on cost savings through early intervention.^1 2^ Refocusing the health system to primary and community care, as well as providing cost-effective services, has been an aim of the UK government for the last decade, with varying degrees of success.^3^ The transfer of eye care from hospital to community settings is not new. It has been widely practised for conditions such as glaucoma and diabetic eye complications.^4 5^ This is an international trend as well. The Finnish and Australian health systems have implemented innovative models of care which share the management of patients with chronic eye diseases between ophthalmologists and optometrists with positive outcomes for health systems including increased access for patients, service efficiency and cost-savings.^6^ Shared care was mostly implemented as a formal programme and funded publicly through the national health insurance, either through direct employment of staff (eg, hospital clinicians), or reimbursement using a fee-for-service (eg, community optometrists). In Australia in particular, shared care between community optometrists and hospital-based ophthalmologists for patients with stable age-related macular degeneration (AMD)^6^ was based on informal arrangements between independent optometrists and ophthalmologists, where funding would come from a mix of patient out-of-pocket (co-payment) fees and government reimbursements.^6^

AMD is the most frequent cause of blindness and accounts for 50% of all certifications of visual impairment in the UK.^7 8^ Neovascular AMD (nAMD) (exudative or wet AMD) is a prevalent, progressive retinal degenerative macular disease mainly affecting the elderly population, causing sudden onset of vision loss. Capacity within hospital-based ophthalmology services for management of nAMD is severely constrained. There is an increasing demand for hospital eye services (HES) which is not being met and continues to grow—currently accounting for nearly 10% of all outpatient appointments and 6% of surgery in the UK.^9^

For many patients with nAMD, the disease will become inactive at some point in their treatment. A key tool in the diagnostic process is the optical coherence tomography (OCT) scan which is a non-invasive imaging test that uses light waves to create detailed pictures of the inside of the eye. OCT scans are used to diagnose and treat eye conditions. The scan produces cross-sectional images of the eye, allowing practitioners to see the retinal layers. Clinical decision-making roles in medical retina services have been greatly helped by the advances in imaging technology, and about two-thirds of departments in the UK interviewed by the Royal College of Ophthalmologists now have non-ophthalmologists making clinical decisions in their AMD pathways. The majority of those were optometrists, but nurse practitioners, nurse consultants, orthoptists and ophthalmic healthcare science practitioners were also involved.^2^

The 2016 ‘Effectiveness of Community vs Hospital Eye Service’ (ECHOES)^9^ showed that there is potential for safe and effective follow-up of patients with quiescent (stable) nAMD (QnAMD) by suitably trained non-medical practitioners such as optometrists. The ECHOES study found that optometrists’ ability to make nAMD retreatment decisions from vignettes was not inferior to ophthalmologists’ ability and proposed that shared care with optometrists monitoring QnAMD lesions has the potential to reduce workload in hospitals. If safe, integrated and quality assured community care can be developed, this should provide opportunities to make services more accessible and convenient for patients while also easing pressure on hospital eye departments and potentially lowering costs.

The ‘Quality-Assured Follow-Up of QnAMD by non-medical practitioners’ (FENETRE) pilot study evaluated the development, delivery, acceptability and evaluation of a modular training programme for community-based, non-medical practitioners monitoring patients with QnAMD. It also explored patient and practitioner acceptability of community-based QnAMD care relative to hospital-based care. Community-based nAMD monitoring by trained optometrists is safe for detecting nAMD disease activity, with a small increase in unnecessary hospital referrals. Community monitoring achieved very high sensitivity (close to hospital-based care), meaning almost all true recurrences were detected. However, this came at the cost of lower specificity, leading to more false positives—patients flagged for suspected recurrence who did not actually need treatment. Findings indicated that the development and implementation of a collaborative community monitoring model is feasible, with satisfaction among community optometrists regarding training and accreditation, and broad acceptance for the pathway by both patients and practitioners. This study builds on prior pilot work. We assess how acceptable community follow-up is for QnAMD when led by trained non-medical practitioners.^10^

## Methods

We will first provide details of the overarching FENETRE randomised control trial, before outlining the specific methods associated with the qualitative process evaluation.

### The FENETRE randomised controlled trial

In the trial, 704 participants with QnAMD were recruited and randomised to either continue hospital-based secondary care or to receive follow-up within a community setting. These patients came from 16 hospital settings and 70 community optometry settings, of which 59 remained active in the follow-up phase of the study. 350 patients were randomised to community care (intervention) and 354 patients to hospital-based care (control). Participants in both groups were monitored for disease reactivation over the course of 12 months and referred for treatment as necessary. Outcome measures assessed the non-inferiority of primary care follow-up accounting for accuracy of the identification of disease reactivation, patient loss to follow-up and accrued costs and the budget impact to the National Health Service.^11^

The process evaluation was conducted independently from the parent trial to ensure independence of findings.

#### Sampling and recruitment for the process evaluation

Six optometry practices operating the QnAMD clinics and six hospitals in the control group were recruited to the qualitative study. Sampling of sites was performed purposively with sites being selected based on geographical diversity and to reflect a range of practice sizes. The purposive sampling method was selected to generate a broad range of views on whether and how the follow-up was acceptable to participants. Additionally, recruitment was restricted to those sites where active FENETRE appointments were taking place in community practices, and where hospital sites were also accessible to the researchers. Optometrists and ophthalmologists were purposively recruited from selected sites based on their knowledge of, and involvement with, the FENETRE trial. Before COVID-19, patients were recruited in clinics where researchers were attending. Researchers were informed of all participants’ appointments in advance by local study research teams. After COVID-19, this process was done remotely. Research administrators, clinicians and nurses asked FENETRE participants if they would be willing to be interviewed. Participants were randomly selected from those taking part in the wider trial, which was limited by researcher availability for each patient’s individual appointment.

We are not reporting specific demographic data to avoid identification, particularly as some of these participants are still under hospital care. We can, however, report that all patient participants were 55 or older as that was one of the trial inclusion criteria. Participants were retired and all sites apart from site 6 were urban.

### Data collection

Semistructured face-to-face interviews before COVID-19 and telephone interviews after COVID-19 were conducted with patients attending either hospital or community QnAMD appointments. The process evaluation team was only sent the details of participants who were willing to be interviewed from the research nurses who recruited them; no information was provided on people who refused to take part in the study. Interviews were also sought with optometrists and ophthalmologists involved in delivering QnAMD care in HES. A total sample of 25 patients (16 randomised to receive follow-up care in the community and 9 in the hospital) and 16 professionals (12 based in the community and 4 based in hospitals) were selected from across the study and control arm (online supplemental files 1-3). Patient interviews were audio-recorded and lasted 15–35 min. Community optometrist interviews were audio-recorded and lasted 15–40 min.

Data were collected by Research Associates SMR and JKC between March 2021 and April 2023. SMR is an experienced qualitative researcher, with recent studies covering eye care and its links to social care, as well as the acceptability of clinical pathways. JKC is a mixed-methods researcher with previous experience in conducting qualitative research in various health-related topics and involving both patients and healthcare professionals. All participants were reassured that the interviews would be confidential to ensure freedom of expression and reduce the risk of bias. No previous relationship existed between researchers and participants ahead of the interview being scheduled and consent being taken. Notes were taken during the interviews and key observations were shared between researchers during regular meetings.

Telephone interviews were conducted as a response to restrictions placed by the pandemic, but this was felt to be less of a risk than not collecting their data. Telephone interviews were not felt to have made a big difference in terms of data collection, though a possible downside of telephone interviews is that the researchers cannot see non-verbal cues, such as body language and facial expression. On the other hand, a positive of telephone interviews is the convenience to the participants which potentially positively influenced recruitment numbers.

### Patient interviews

Open-ended interview schedules were developed to investigate how patients access clinics and their views on being seen in either setting, any changes they would make to clinic organisation, whether staffing and frequency of appointments were adequate and their views of care received (online supplemental file 4). Questions were oriented to perceptions of what it meant in terms of time, travel, parking and quality of care to visit a community clinic or hospital for routine follow-up.

### Community optometrist interviews

For the community optometrist interviews, participants were invited to semistructured interviews. Telephone interview schedules explored participants’ views on the FENETRE intervention and any elements they would change, the extent to which their practice was reorganised to accommodate FENETRE appointments, and any impacts on service delivery to other patients. Additionally, optometrists were asked whether they felt the FENETRE pathway would achieve its aim of managing QnAMD in community care, taking into account patient safety, outcomes, experience and access to care.

### Hospital-based practitioner interviews

Interviews were also sought with optometrists and ophthalmologists involved in delivering QnAMD care in HES. Interview schedules covered practitioner perspectives of QnAMD service delivery in their setting, as well as views on the FENETRE pathway and potential barriers to its implementation.

### Data analysis

All researchers (SMR, JKC and SV) independently coded the transcripts using the framework developed by SMR for the FENETRE pilot^10^ and double-checked the coding with Professors Aled Jones, Heather Waterman and Molly Courtenay (the last two colleagues retired during the course of the study). Qualitative data were organised in NVivo (QSR International, qsrinternational.com) with a deductive thematic framework analysis approach adopted.^12^ This aligned with the principles of Braun and Clarke^13^ and followed steps of familiarisation of the research team with the data, generating initial codes and themes, then reviewing and defining these. Initially, the Theory of Acceptability framework^14^ was a starting point guiding the analysis for this paper, though this evolved as data were collaboratively discussed. To do this, regular meetings were held between SV, SMR and JKC reviewing data interpretation, as well as revising and adapting the final thematic framework (online supplemental file 5). This took multiple iterations, with four monthly meetings held over the course of the analysis, alongside informal discussions between researchers and senior members of the team. Once data saturation was felt to be reached and new codes were no longer identified in the sample, discussions advanced towards the organisation of themes and understanding the relationships between them. Our definition of data saturation refers to the state in the analysis where no new information, themes or insights emerge from the data in relation to the acceptability of the trial. This was achieved through discussions between the researchers during the data analysis phase as well as during the period when the paper was being developed. Again, framework analysis was used to map these connections between themes, identifying the ways in which community-based QnAMD clinics were acceptable for patients and professionals, as well as any perceived issues or concerns. SV led on the analysis for this paper and the communication between co-authors in different institutions. SV mapped the codes to Theory of Acceptability constructs and identified illustrative quotes per theme for this paper in discussion with the other researchers. As the data did not match directly all seven constructs of the Theory of Acceptability, the authors decided to structure the paper around the different factors affecting acceptability for each participant group as the arguments were better supported when triangulated with quotes from different participant groups. The proposed plan was reviewed by and agreed with parent trial leads (KB and AS). Reporting followed the Consolidated criteria for reporting qualitative research (online supplemental file 5).

## Results

A total of 21 themes were identified in relation to the acceptability of community follow-up, material aspects of care and organisation of shared eye care services (online supplemental file 6 and sample quotes in file online supplemental file 7,8). The data presented here cover all 21 themes organised in five broad categories (Acceptability of community care monitoring, The physical environment, The organisation of care, Issues specific to patient experience and Issues specific to staff experience). Full details are presented in online supplemental table 6. In this paper, we present the most pertinent issues raised by patients and by staff in relation to the acceptability of community care. These are broken down into three sections: General Acceptability themes, Patient Acceptability Themes (Patients &and Healthcare Professional Perspectives) and Healthcare Professional Acceptability Themes (Healthcare Professional Perspectives only).

### General acceptability of FENETRE

Community care for QnAMD was widely acceptable for both groups of patients (those receiving community and hospital-based care) as well as eye care professionals. Professionals working within both settings found that the FENETRE pathway was a desirable task for the profession to be undertaking.

You know, patients like it. I think it’s good for our profession. It upholds our professional standards, and so for those of us who are interested in that sort of clinical care … then I think it’s a great thing to do. (community optometrist 1–007)

Community optometrists were happy with the training they received and did not report any issues in delivering follow-up care. Professionals based in the hospital also felt that it was acceptable for community optometrists to be undertaking the follow-up and for patients not to be visiting the hospital for such appointments (see online supplemental table 7).

Patients expressed confidence in community optometrists, were overall satisfied with the care that they received, its organisation and the communication with staff.

It’s well organised. I get good communication. They treat me…various people who contact me, with the utmost respect. (patient receiving care in the community 4-008)

Furthermore, they particularly appreciated the clinic potentially being closer to their home and the reassurance that they could have a hospital appointment should they need to have injections (online supplemental file 7). Overall, they did not feel that follow-up care in the community was of a lesser standard than the follow-up care in hospital.

### Factors affecting patient acceptability

Patients and professionals both mentioned transport and location, the physical environment and patient mobility, appointment characteristics and patient–healthcare professional interactions. In this section, we report data covering these themes from professional and patient cohorts set across both hospital and community settings.

#### Appointment characteristics and patient–healthcare professional interactions

Community-based QnAMD appointments offered the same core structure as hospital-based appointments. Some healthcare professionals felt that the community setting allowed more time for consultation and opportunities for patients to ask questions:

In the hospital environment … often they simply don’t have time … my patients will have been to a hospital appointment, and then will want to speak to me about it afterwards … “Well, I didn’t have any opportunity to ask any questions…” (community optometrist 1-007)

Patients also reported that they valued the level of focus and attention that the longer community appointments afforded them:

He was so professional … he reviewed the cross section through my eye and the back there. That was so important to talk about, because I was aware of that… from the eye hospital (patient receiving care in the community 2-036).

Professionalism was valued during interactions between patients and community optometrists. Alongside this, though, some patients were familiar with community practices and professionals, having had their primary eye care carried out in that setting. Professional and patient participants felt that this could remove barriers to interactions and seeking care:

I think they felt quite happy coming along because … they know how the practice works. They know me, and yeah, I think it just made me feel a bit more comfortable. It wasn’t like a big deal for them to come in.’ (community optometrist 2–005)

Overall, patients reported receiving clear instructions (2-028), finding the visits enjoyable (2-005) and having confidence in the optometrist (2-036). Logistical aspects, such as booking or amending appointments, were also referenced as being valued by patients:

They always say, if you have any problems … you ring up and you come down see us. You don’t have to wait those six, seven, eight weeks. If there’s a problem in between each appointment, they always say, ‘Give us a ring and we’ll see you.’ (patient receiving care in the hospital 2-027)

While appointments in both hospital and community settings shared the same technical and clinical characteristics, the community setting was generally seen as enhancing the potential for patient and professional interactions. This was particularly heightened where patients were already familiar with either the community practice or optometrist.

### Transport and location

Professionals in the community and the hospital felt that the community QnAMD appointments would often be better located for patients than the hospital setting, and therefore might be easier to attend.

Optometry practices are well placed to see patients … often the patient’s not going to have to travel a long way, and it’s much easier to get to. (community optometrist 2-005)

It [the hospital] is a little bit out in the city. So some patients do have to get two buses, or rely on family and friends to bring them.’ (hospital nurse 3-002)

One patient receiving follow-up care in the community reported that some hospital sites were difficult to get to and they relied on lifts from taxi drivers or people with caring roles:

I have somebody that helps me and she has a car so she takes me. If not, I get a taxi, and it’s extremely easy as opposed to the [name of hospital] which is absolutely unbelievable and difficult to get to (patient receiving care in the community 3-011)

Another patient also reported that parking is an issue in the eye hospital so they made arrangements to be dropped there by their spouse and collected after the appointment:

My wife can drive me and then coming out, she can join me home. So the only bad thing is you can go shopping! (patient receiving care in the hospital 2-028)

Some patients attributed preference for follow-up in the community because it reduces commuting time:

I would say one of the biggest benefits, is the…you know, accessibility of [community optometry practice] as compared to [xxx] hospital (patient receiving care in the community 3-011)

Some patients randomised to receive follow-up in the hospital expressed preference for their care to remain in the hospital, attributing familiarity with the location of care as a reason.

You know, I’m not saying that I wouldn’t trust anyone else I can… but I’ve got… I’ve got used to going in there, and you know, I find the routine of timing… I know when to… how to get there and when to get there for. (2-027 – patient receiving follow up in the hospital)

I prefer the hospital, because there were less people there and you didn’t have to go through town … And I don’t know, the distance was similar. The hospital was more… well, it’s a hospital environment, whereas [the community optometry practice] … it’s the busiest optician I think I’ve ever been in. (6-001 patient receiving hospital treatment)

Proximity of the setting and familiarity with the route appeared to be a primary consideration for patients, with some reporting that they would not mind attending either setting if it was easy to reach. This was interlinked with other issues such as the level and frequency of public transport, or the availability of carers to help get patients to their appointments.

### The physical environment and patient mobility

Issues around the physical environment and patient mobility were mentioned in several interviews. While community practices were often easier for patients to get to, some practices operated in older, sometimes grade II-listed, buildings. For some of these, accessing equipment such as OCT would involve the use of stairs, with there being no lifts or escalators available:

The only other sort of slight issue that we have at [community optometry practice] is that the OCT machine is upstairs and there’s nothing available downstairs. […] if people were to come into community care in the longer term, there’d have to be some arrangements for having an OCT downstairs to enable access. (community professional 4-004-FU)

They do some of the tests upstairs. Stairs are very steep, and now, that doesn’t bother me at the moment. It may do in five, ten years’ time. I could imagine an older, less able person might find that difficult. (patient receiving care in the community 4-008)

Oh no, no, no. There was no lift. It was […] going upstairs like a maisonette. (patient receiving care in the community 1-066)

Reportage of this issue was limited to one or two practices within the broader sample, with most being fully accessible. Nevertheless, given there can be comorbidities between mobility issues and QnAMD for older people, this should be factored into where patients are asked to attend their appointments.

### Factors affecting healthcare professional acceptability

As stated previously, there was broad acceptability for the FENETRE pathway from professionals in both hospital and community settings. That said, three topics were reported by professionals as potentially influencing this. These related to the adequacy of the FENETRE training for community optometrists, the technological infrastructure supporting hospital-to-community communication such as the transfer of OCT scan data, the costs associated with delivering QnAMD in the community beyond the end of the FENETRE trial, and, relatedly, the need for further granularity in how community practices were defined, for example, whether they were independent or chain practices.

#### FENETRE training

We have previously reported on the provision of FENETRE training for optometrists looking to provide QnAMD appointments in the community.^10^ Largely, the training was perceived as being adequate for optometrists to successfully complete appointments and gave optometrists confidence in using skills that may have previously been unfamiliar to them.

It improved my knowledge of things, obviously. You know, OCT is not something that we’re sort of specifically trained in … for me, it was a challenge to sort of improve my skills, improve my knowledge, and from that perspective I think, you know, it did help in that respect. (community optometrist 1-006)

However, other optometrists reported being familiar with using the OCT prior to attending FENETRE training.

They felt that the people that were doing this had familiarity with the OCT and because the modules were quite in-depth and the ones on OCT and the macular, they felt that was enough. (Community healthcare professional 3-001)

The upskilling of staff to perform QnAMD monitoring in the community was felt to be sufficient by all professionals recruited to the process evaluation. As noted in previous articles, engagement with real-world OCT scans and scenarios was felt to be particularly valuable in ensuring practitioners felt confident when carrying out FENETRE appointments.

#### Data transfer and technological infrastructure

The data transfer system used for FENETRE was developed in-house at Moorfields by the Research & Development Information Technology (R&D IT) team. The front end used a bespoke Microsoft Visual Studio application and the back end (data storage) was hosted on Moorfields Research Database SQL servers. Local hospital trial coordinator and local optometry staff managed uploading both image and patient data (case report forms, CRFs) on a monthly basis on a bespoke electronic Case Report Form (eCRF). This was to be done using Moorfields remote log-in tokens, where the users logged onto clinical services and saw the local drives of the PC they were using. The database will be validated to Good Automated Manufacturing Practice (GAMP) 5 standards.

Several professionals mentioned there were pre-existing issues in data transfers between hospital and community settings which also affected some elements of FENETRE-related file-sharing. These were specific to the transfer of large data files, such as full volume OCT scans, which were often transferred as still images. For those experiencing these issues, this could limit their capacity to fully assess patients:

It’s great to have the scans on the online portal, but … you’re only getting like a section or two of what that matter looks like… it kind of makes it a bit more difficult to assess if it’s active or not active (community optometrist 2-006)

One of the disadvantages on both sides of not being able to transfer the full OCT file is that I’ve had a few patients that I’ve seen for the first time and I’ve really not got anything to go off a few sort of grainy black and white photocopied versions (community optometrist 5-001FU)

Comparing historic and current OCT scans is a core part of the diagnostic process for QnAMD. As such, some community appointments where comparison was not possible saw patients referred back to the hospital, mitigating any patient safety issues. However, where comparison was possible, optometrists reported greater confidence in ongoing monitoring of QnAMD.

#### Cost of delivering QnAMD follow-up care

Professionals were asked about the sustainability of delivering QnAMD follow-up care in the community beyond the duration of FENETRE, with this resulting in a mixture of views. For some, it was felt that the financial remuneration associated with appointments may not fit within some community practice business models:

If I had to spend 40 minutes with … all of these patients for, what is it, £48 or something…? You know, you can’t run a practice on, what’s that, £60 an hour, isn’t it? It doesn’t work. (community optometrist 1-008)

If you look at the remuneration … it probably doesn’t even cover the… chair time as it stands… there’s two ways that opticians could look at it. One is that it’s… if they didn’t have a full appointment diary, then it’s… you know, it’s extra income. But assuming that they did have a full appointment diary, the only advantage that they get out of it is… is to have a retained patient in their… practice. (community optometrist 4-004FU)

Some professionals noted that there was nuance around which community practices might be more likely to incorporate FENETRE appointments successfully. Particularly, whether a practice was part of a larger chain of stores or independent was mentioned as a potentially influential factor:

A lot of the sort of chain practices tend to be maybe shorter appointment times and very busy. So I think it might be harder for them to fit in study patients. And also, just sadly, the model of optometry in the UK is basically you don’t very much have an appointment, but you want people to buy glasses, and that’s how you make your money. (community optometrist 2-005)

Another professional mentioned that it was difficult to find the time to complete the administration that is required for each appointment during the working day: *if you’ve got a busy diary full of patients, and you know, no free time in the day, this is just stuff that goes into the evening or lunchtime* (community optometrist 5-001FU)

Ultimately, issues around financial sustainability will be central to broader acceptability of FENETRE appointments in real-world community settings. Study data suggested that some professionals saw ongoing community monitoring as incompatible with their business models. This was felt to particularly be the case with larger chain stores, though future research could help to further develop and understand this theme.

## Discussion

This process evaluation explored the acceptability of community-based QnAMD monitoring for both patients and healthcare professionals. Key themes influencing patient acceptability were transport and location, appointment characteristics, patient–healthcare professional interactions, and the physical environment, particularly for patients with mobility issues. Themes mentioned by healthcare professionals were satisfaction with the FENETRE training received by community optometrists, concerns regarding data transfer and technological compatibility between the IT systems used by HES and community optometrists, and the costs involved in the administration of delivering QnAMD follow-ups in the community. Overall, the process evaluation showed that community care for QnAMD was widely acceptable among patients and health professionals but not preferred to hospital-based follow-up care. Data on financial sustainability were inconclusive. This complements the main trial findings that showed that community monitoring is not inferior to hospital monitoring.^15^ This data consolidates the findings of the main trial which concluded that community-based nAMD monitoring by trained optometrists is safe for detecting nAMD disease activity, with a small increase in unnecessary hospital referrals. The study’s methodological approach may offer a transferable framework for evidence-generation in shared-care clinical models, easing hospital demand and improving access without compromising safety.^15^

Hospital sites were not reported to be inaccessible, but often further away from patients’ homes and often difficult to get to. These issues were exacerbated when patients could not drive or walk to their appointments with ease. Community opticians were reported to often be closer to patients’ homes. Patients often relied on family members and taxi drivers to provide them with a lift to the location of their follow-up care appointment and proximity to one’s home was a key factor affecting acceptability irrespective of the location of care. Savings in travel time were also reported favourably by patients accessing shared eye care services in Australia.^16^ Similarly, in the USA, distance and drive time are concerns for patients who have limited access to care, especially patients from low-resource communities.^17^

Patients randomised into community care practices reflected positively on their care, highlighting such factors as enhanced patient–practitioner interactions, as well as potential for increased convenience. Patients also reported that community optometrists were well-trained and polite. Both patients and community optometrists mentioned that familiarity with the community optometry practice and/or the optometrist through previous eye care engagements contributed to positive experiences when accessing QnAMD follow-ups in the community. The positive impact of effective communication with older adults on patient-centred outcomes has been reported in the literature on patient-centred health outcomes.^18^

Some patients highlighted that community practices varied from hospital settings, particularly in relation to accessibility. For instance, some practices were unable to offer lifts to upper floors, which may be impractical for those with mobility issues. Access barriers in the built environment have been widely reported in the literature on disability and health inequalities.^19 20^ The role of the built environment for healthy ageing has received increased attention in research and policy. Older adults may be particularly susceptible to barriers in their local built environments, especially when they experience declines in functional capacity and are in need of accessible transportation and services.^21^ It was also noted that some participants randomised into the hospital group of the study expressed preference for aspects of the hospital setting. This was often without having experienced community-based QnAMD care and was largely felt to be attributed to a passive acceptability of, or familiarity with, hospital-based eye care. This was consistent with previously reported findings from the pilot study^10^ and suggests there may need to be reassurance for older patients who are allocated to community care if implemented on a wider scale. Issues around accessibility, particularly with regard to older people, may require further investigation so as to comply fully with the Equality Act 2010.^15^

No data collected during the FENETRE study indicated concerns from hospital practitioners relating to the capabilities of community optometrists. This could potentially be attributed to the effectiveness of the FENETRE training; however, we only interviewed four hospital staff and only one of them was an ophthalmologist. So there could have been issues that did not come across in the process evaluation. Several professionals mentioned there were issues in data transfers between hospital and community settings. These were specific to the transfer of large data files, such as full volume OCT scans, which were often transferred as still images. Even though these issues have not been explicitly reported in the shared eye care literature, issues with poor electronic health record system interoperability are well-known issues in the use of health information technologies in most high-income countries worldwide.^22^

Another concern for professionals was the cost of delivering specialised follow-up care in the community. This is due to the economic viability of community optometry practices being highly dependent on dispensing glasses. There were nuances in how FENETRE was perceived by community care practices based on their size, with larger practices potentially seeing less benefit than smaller independents. We found that the cost of delivering QnAMD follow-up care in the community beyond the duration of the trial was a cause for concern. The real-world acceptability will hinge on whether optometry practices would be able to accommodate the appointments and associated workloads. Similar issues around time constraints as a barrier to long-term acceptability have been reported elsewhere and will need consideration going forward.^1^ Our findings support the realist assessment of shared eye care models for chronic disease which argued that scalability of shared eye care will require investment from broader health systems to support financial incentives, motivate providers and integrate information systems.^6^ However, this paper only reflects the qualitative process evaluation findings; the main economic analysis of the parent trial will be published at a later date.

### Limitations

The following limitations have been identified:

- The trial was suspended during COVID-19 for 102 days and this disrupted recruitment to the process evaluation. Due to the large size of the trial, there might have been issues that were not encountered. However, the reports from the 25 patients across 6 sites offer rich and valuable insights into how the intervention was received across cohorts. Nevertheless, not tracking refusals limits assessment of selection bias.
- Some of the data collection took place during COVID-19, where opportunities for face-to-face interviews and observations were limited. Even though telephone interviews were found acceptable by patients, they may undercapture non-verbal cues.
- There was limited recruitment of hospital professionals, meaning that interpreting any issues around perceptions of community optometry in that setting is difficult and requires further exploration in future studies.

### Recommendations for future research

Further research is needed on the financial sustainability of delivering QnAMD care in the community and exploring the capacity of different types of optometry practices to take on long-term QnAMD follow-up appointments. Issues of equity and access could be further explored if community follow-up is taking place in older buildings. Finally, further research could explore issues regarding the technological compatibility between the hospital and the community eye care settings and how to mitigate any impact on the diagnostic process.

## Conclusions

Overall, the process evaluation showed that community care for QnAMD was widely acceptable among patients and health professionals but not preferred to hospital-based follow-up care. Specifically, patients randomised to the community found the community optometry-led QnAMD follow-up acceptable. Some patients randomised to the hospital showed preference for QnAMD to take place in the hospital. Proximity of the follow-up location to one’s home was a big component of acceptability (irrespective of whether that was a hospital or a clinic). However, building accessibility of some community optometry practices was reported as potentially problematic. Professionals based in HES found the community QnAMD acceptable. Professionals in the community felt that the FENETRE training covered the skills required to perform QnAMD follow-up. Some concerns were expressed about the financial sustainability of delivering QnAMD follow-up in community optometry practices after the end of the trial, such as financially supporting the administration and record-keeping workload. Technological compatibility issues between the IT systems of the hospital and the clinic were reported in relation to OCT image transfer. These issues were mitigated by good communication between professionals. To address these issues, future studies of shared eye services could consider exploring further the full cost of follow-up care, interoperable IT agreements, periodic refresher training, audit and feedback, and minimum accessibility standards for practices. These topics could be taken into consideration by both academics designing shared eye service trials and practitioners designing new service pathways.

## Supplementary material

10.1136/bmjopen-2025-101379 online supplemental file 1

## Data Availability

No data are available.
