# Supplementary material for: Comparing community-based monitoring to hospital-based care of patients with quiescent age-related macular degeneration: a qualitative study of patient and practitioner perspectives on acceptability and access
Source: BMJ Open. 2026 Feb 4;16(2):e101379. doi: 10.1136/bmjopen-2025-101379 (PMC12878339; doi:10.1136/bmjopen-2025-101379)
Supplement: online supplemental file 1 [file bmjopen-16-2-s001.docx]

# Supplementary files

## Supplementary file 1

| **SITE** | **Community Patient** | **Hospital Patient** | **Community Optom** | **Hospital HCP** | **TOTAL** |
| --- | --- | --- | --- | --- | --- |
| 1 | 3 | 1 | 3 | 0 | 7 |
| 2 | 1 | 2 | 2 | 0 | 5 |
| 3 | 3 | 2 | 2 | 1 | 8 |
| 4 | 3 | 1 | 2 | 1 | 7 |
| 5 | 3 | 2 | 2 | 1 | 8 |
| 6 | 3 | 1 | 1 | 1 | 6 |
| **Total** | 16 | 9 | 12 | 4 | 41 |

Table 1: Distribution and characteristics of interview participants

## Supplementary file 2

| **HCP interviews** | | |
| --- | --- | --- |
| **Ref.** | **Setting** | **Job role** |
| 5-002 | Community | Healthcare professional* |
| 1-006 | Community | Optometrist |
| 1-007 | Community | Optometrist |
| 1-008 | Community | Optometrist |
| 2-005 | Community | Optometrist |
| 2-006 | Community | Optometrist |
| 3-001 | Community | Healthcare professional* |
| 5-005 | Hospital | Optometrist |
| 3-002 | Hospital | Senior Ophthalmic nurse |
| 3-003 | Community | Optometrist |
| 5-001FU | Community | Optometrist |
| 4-004FU | Community | Optometrist |
| 4-005 | Community | Optometrist |
| 4-006 | Hospital | Healthcare professional* |
| 6-001 | Hospital | Ophthalmologist |
| 6-002 | Community | Optometrist |

* The healthcare professional description has been attributed where we only have healthcare professional are the ones where we didn't know what they were. Their site file notes only mention healthcare professional.

## Supplementary file 3

| **Patient interviews** | | |
| --- | --- | --- |
| **Ref.** | **Setting** | **Sex** |
| 1-066 | Community | Male |
| 1-076 | Community | Female |
| 1-082 | Community | Female |
| 2-027 | Hospital | Female |
| 2-028 | Hospital | Male |
| 2-036 | Community | Male |
| 3-011 | Community | Female |
| 3-019 | Hospital | Female |
| 3-020 | Hospital | Male |
| 5-025 | Hospital | Female |
| 5-027 | Hospital | Male |
| 5-035 | Community | Male |
| 5-037 | Community | Female |
| 4-007 | Hospital | Male |
| 4-008 | Community | Male |
| 4-010 | Community | Female |
| 1-117 | Hospital | Female |
| 4-015 | Community | Female |
| 5-068 | Community | Male |
| 6-001 | Community | Female |
| 6-002 | Hospital | Male |
| 6-003 | Community | Male |
| 6-004 | Community | Female |
| 3-999 | Community | Male |
| 3-998 | Community | Female |

## Supplementary file 4. Qualitative interview schedules

### Healthcare professionals

**General Appointment Issues**

1. How did you feel your consultations with Participant are going in the clinic today?
2. Tell me about what happened during the appointment of XXXX from your perspective…

- Prompts: Initial discussion; eye tests; outcomes.

1. You said XXXXX during the appointment. What were you trying to find out?
2. Why was that important to you?
3. Do you feel you had this issue resolved?
4. Can you think of anything else about the care provided for Participant with nAMD you would like to have changed?
5. If so, how would this have improved the care?

Prompt: access, organisation, staffing, frequency of appts, resources, training

1. How did you feel about the care at the end of the appointment?
2. Can you think of any other issues that could improve the care you gave today? If so, how would you able to overcome these issues?

### Patients

General Appointment Issues

1. How did you feel your appointment in the clinic went today?

2. Tell me about what happened during the appointment from your perspective…

- Prompts: Initial discussion; eye tests; outcomes.

3. Did you understand what the doctor* was telling you?

a. When the doctor was mentioning XXXXX did that all make sense?

b. Was there anything you would have liked to ask the doctor that you didn’t?

c. Could anything have been made clearer for you?

4. You said XXXXX during the appointment. What were you trying to find out?

a. Why was that important to you?

b. Do you feel you had this issue resolved?

5. Can you think of anything else about the appointment you would like to have changed?

a. If so, how would this have improved the appointment?

Prompt: access, organisation, staffing, frequency of appointments

## Supplementary file 5. Consolidated criteria for reporting qualitative studies (COREQ): 32-item checklist.

| No | Item | Guide questions/description | Reported on section |
| --- | --- | --- | --- |
| Domain 1: Research team and reflexivity |  |  | Data analysis |
| Personal Characteristics |  |  | Data collection |
| 1. | Interviewer/facilitator | Which author/s conducted the interview or focus group?    Simon Read and Judit Csontos | Methodology> Data collection |
| 2. | Credentials | What were the researcher's credentials? *E.g. PhD, MD*    SV: BSc, MSc, PhD  SR: BA, MA PhD  JC: BSc, MSc, PhD  AJo: RN, PhD  AS: BSc Optom, MSc  AJ: BSc  KB: MB BS, MD(Res), FEBO, MRCOphth, FFCI | Author list |
| 3. | Occupation | What was their occupation at the time of the study?    SV*:* Research Fellow  JC: Research Associate  SR: Research Associate  AJo: Professor  AS: Specialist Optometrist  AJ: Trial Manager  KB: Professor | Author affiliations |
| 4. | Gender | Was the researcher male or female?    SV: female  JC: female  SR: male | Author affiliations |
| 5. | Experience and training | SV*:* qualitative health services research  SR: qualitative health services research  JC: health services research, systematic reviewing  AJo: healthcare delivery and organisation  AS: optometry  KB: ophthalmology, clinical trials | Author affiliations |
| Relationship with participants |  |  |  |
| 6. | Relationship established | Was a relationship established prior to study commencement?    No relationship was established prior to the study commencing. Participants were recruited from clinics. | Data collection |
| 7. | Participant knowledge of the interviewer | What did the participants know about the researcher? e*.g. personal goals, reasons for doing the research*    Participants did not have prior knowledge of the researcher profiles. They were informed about the research through the Participant Information Sheet. | Data collection |
| 8. | Interviewer characteristics | What characteristics were reported about the interviewer/facilitator?*Brief intro with reasons and interests in the research topic*    Same as in 7. | Data collection |
| Domain 2: study design |  |  |  |
| Theoretical framework |  |  |  |
| 9. | Methodological orientation and Theory | What methodological orientation was stated to underpin the study?    *Framework analysis and Theory of Acceptability* | Data analysis |
| Participant selection |  |  |  |
| 10. | Sampling | How were participants selected?    *Research administrators, clinicians and nurses asked FENETRE participants if they could be willing to be interviewed.* | Data collection |
| 11. | Method of approach | How were participants approached?  *Same as 10* | Data collection |
| 12. | Sample size | How many participants were in the study?  41 | Interviewing process |
| 13. | Non-participation | How many people refused to participate or dropped out? Reasons?  *We do not have information on how many people refused to take part in the study. We were only sent the details of participants who were willing to be interviewed from the research nurses who recruited them.* | Interviewing process |
| Setting |  |  |  |
| 14. | Setting of data collection | Where was the data collected?    Over the phone. | Interviewing process |
| 15. | Presence of non-participants | Was anyone else present besides the participants and researchers?    *No* | Interviewing process |
| 16. | Description of sample | What are the important characteristics of the sample?    *TBC* |  |
| Data collection |  |  |  |
| 17. | Interview guide | Were questions, prompts, guides provided by the authors? Was it pilot tested? | Appendix |
| 18. | Repeat interviews | Were repeat interviews carried out? If yes, how many?  *No* | Data collection |
| 19. | Audio/visual recording | Did the research use audio or visual recording to collect the data?  *Audio* | Data collection |
| 20. | Field notes | Were field notes made during and/or after the interview or focus group?  *Yes, during and after but not used in formal analysis* | Data collection |
| 21. | Duration | What was the duration of the interviews or focus group?  *Range: 26 to 70 minutes, with the majority lasting for one hour.* | Data collection |
| 22. | Data saturation | Was data saturation discussed?  *Yes* | Data collection |
| 23. | Transcripts returned | Were transcripts returned to participants for comment and/or correction?  *No, but the themes and findings were for a member check* | n/a |
| Domain 3: analysis and findings |  |  |  |
| Data analysis |  |  |  |
| 24. | Number of data coders | How many data coders coded the data?  *3* | Data analysis |
| 25. | Description of the coding tree | Did authors provide a description of the coding tree?  *yes* | Appendix |
| 26. | Derivation of themes | Were themes identified in advance or derived from the data?  *Derived from data* | Data analysis |
| 27. | Software | What software, if applicable, was used to manage the data?  *NVivo* | Data analysis |
| 28. | Participant checking | Did participants provide feedback on the findings?  *No* | Data analysis |
| Reporting |  |  |  |
| 29. | Quotations presented | Were participant quotations presented to illustrate the themes / findings? Was each quotation identified?  *yes* | Results |
| 30. | Data and findings consistent | Was there consistency between the data presented and the findings?  *yes* | Results |
| 31. | Clarity of major themes | Were major themes clearly presented in the findings?  *yes* | Results |
| 32. | Clarity of minor themes | Is there a description of diverse cases or discussion of minor themes?  *yes* | Appendix |

## Supplementary file 6. Qualitative analytical framework & Links to Theory of Acceptability Framework (Sekhon at al. 2017)

| **Bucket theme/**  **Domain summary** | **Themes** | **Coding frequency** | **Theory of Acceptability domain exhibited in the theme*** |
| --- | --- | --- | --- |
| Acceptability of community care monitoring | Variations or similarities in practice | 31 files  153 refs | Affective attitude |
|  | Patient acceptability | 28 files  192 refs | Affective attitude  Intervention coherence  Perceived effectiveness  Self-efficacy |
|  | Healthcare professional acceptability | 14 files  192 refs | Affective attitude  Burden  Ethicality  Intervention coherence  Perceived effectiveness  Self-efficacy |
|  | Patient HCP interactions | 32 files  145 refs | Affective attitude |
| The physical environment | Technology issues | 16 files  29 refs | Self-efficacy |
|  | Physical environment | 14 files  27 refs | Self-efficacy |
|  | Mobility issues | 13 files  16 refs | Self-efficacy |
| The organisation of care | Busyness of setting | 20 files  41 refs | Burden |
|  | Appointment Structure and timing | 32 files  170 refs | Burden |
|  | Alternate pathways suggestions | 22 files  52 refs | Burden |
|  | COVID-19 | 26 files  45 refs | Burden |
|  | Cost of Care | 10 files  23 refs | Burden |
|  | Purpose of FENETRE | 15 files  26 refs | Perceived effectiveness |
| Issues specific to patient experience | Patient referral or history | 18 files  58 refs | NA |
|  | Transport and Location | 29 files  105 refs | Affective attitude  Self-efficacy  Opportunity costs |
|  | Other Patient Sight or Health issues | 18 files  63 refs | Affective attitude |
|  | Patient understanding of condition | 23 files  92 refs | Ethicality  Intervention coherence |
|  | Patient outcomes | 29 files  92 refs | Ethicality  Intervention coherence |
| Issues specific to staff experience | FENETRE training | 13 files, 27 refs | Affective attitude  Self-efficacy |
|  | Clinical procedure issues | 20 files  58 refs | Affective attitude  Self-efficacy  Opportunity costs |
|  | Staffing | 20 files  58 refs | Affective attitude  Self-efficacy  Opportunity costs |

*The Theory of Acceptability Framework is based on seven constructs:

Affective attitude> How an individual feels about the intervention

Burden> The perceived amount of effort that is required to participate in the intervention

Ethicality> The extent to which the intervention has good fit with an individual's value system

Intervention coherence> The extent to which the participant understands the intervention and how it works

Opportunity costs> The extent to which benefits, profits or values must be given up by engaging in the intervention

Perceived effectiveness> The extent to which the intervention is perceived as likely to achieve its purpose

Self-efficacy> The participant's confidence that they can perform the behaviour(s) required to participate in the intervention

## Supplementary file 7 – Participant quotes exemplifying acceptability

| **Code: Acceptability - Professionals** |
| --- |
| In the community I think there’s a lot of optometrists ... who’d be, you know, interested and willing ... take on some of these sorts of patients. So I think it’s a great idea. (5-002 community) |
| I feel like consultations have been going very well. The patients are arriving, and they feel quite grateful for ... having a follow-up care. (2-006 community) |
| Providing the opticians have the appropriate training and the appropriate scanning equipment, I don’t see a problem with that at all. I think it’s a very good idea, because it would free up our capacity. (3-002 hospital) |
| I think the less time we need to bring people to hospital, the better, particularly in light of the recent pandemic. (5-005 hospital) |
| Yeah because I’ve got rapport with that patient as well, you know, from previous times, so I’d rather see them myself [...] I already had a bit of a relationship with the patient in terms of them coming quite regularly to the store previously, so I kind of knew that this patient would appreciate that (healthcare professional in the community 3-001) |
| **Code: Acceptability - Patients** |
| But he was so professional... I was very confident in what he said....I was very pleased about that. (2-036) |
| As long as the optician was able to interpret ... the scan, I can't see any difference really. … it is taking a bit longer because of COVID, but it's still pretty good, to be honest. It's a lot better than the eye hospital, put it that way. (2-028) |
| I was completely satisfied, you know ... If these people are fully trained and they knew what they were doing, I don’t mind, because I would think what I have done in the eye hospital an optician could do.( 5-027) |
| I’d be quite happy [for my care to be maintained in a community optometry practice], but if I need the injections, obviously I’m going to have to go back to the hospital, which I don’t mind, you know? (4-015) |
| *Well, the time element it’s nil there. […] And when you get there, you’ve got an appointment and you’re there in five minutes (patient receiving care in the community 5-035)* |

## Supplementary file 8 – Exemplary quotes per theme

| Theme | Quotes |
| --- | --- |
| Variations or similarities in practice | *I’d be quite happy going there. I'd be quite happy going up to [the hospital]. I’m quite happy going anywhere as long as I can see. (patient, site 3)* |
| Busyness of the setting | *Having worked in... in the hospital environment, I'm... I'm, you know, very conscious often they simply don't have time, and I... I find that quite a lot, you know, of my patients will have been to a hospital appointment, and then will want to speak to me about it afterwards, because they... you know. “Well, I didn't have any opportunity to ask any questions, so, you know, would... would you ask Polly to give me a call, so that she can just explain to me what they're doing?”, you know. So I think... I think it's… yeah, the... the nature of the beast. We... we choose to spend more time with our patients. I mean, I... I'm... I'm allowing an hour to see FENETRE patients. Part of that, you know, is because of COVID. (healthcare professional, site 1)* |
| Appointment structure and timing | *“They always say, if you have any problems, or anything, you know, that you're worried about, you ring up and you come down see us. You don't have to wait those six, seven, eight weeks. If there's a problem in between each appointment, they always say, 'Give us a ring and we'll see you.'” (patient, site 2)* |
| Staffing | *I: Would you be happy to have these kinds of appointments there?*    *P: I would. I would, yes. I mean, if these people are fully trained and they knew what they were doing, I don’t mind, because I would think what I have done in the eye hospital an optician could do, because when I have my eyes tested for glasses, they do just the same sort of thing, and I presume they’re doing a scan at the back of my eyes, because that’s how my optician realised that there was something wrong with my eyes to start with. (patient, site 5)* |
| Technology issues | *The biggest thing is, it's great to have the scans on the online portal, but you're only getting one... one section. You're only getting like a section or two of what that matter looks like. So it's kind of sometimes a bit harder to just… it kind of makes it a bit more difficult to assess if it's active or not active where I guess if I was in a hospital setting, I would have access… a complete access for that last scan and then people to slice through different sections, see what it was say a month ago or whatever and then... but obviously, in this, it's... it's better than nothing, but it's... it's still quite a significant... significant compromise to feed on because it might have reactivated in a slightly different part, or it might, you know, maybe slightly... slightly unusual architecture in one area. (patient, site 2)* |
| Patient HCP interactions | *I never have to worry. Oh, my goodness, I've got... today my eyes aren't so good. Shall I ring them? You know, but some days they are more blurred than others, but that's how they are. You know… I’ve… I've got the eye drops, which I have, one I have once a day and the other I have two drops a day, morning and night. (patient, site 2)* |
| Clinical procedure issues | *From a clinical aspect, I’m sure for the… for the Eye Hospital, they would far rather we know that all the technology is there to be able to just load up the whole OCT scan. That would be much better for the Eye Hospital to be able to review. (healthcare professional, site 2)* |
| Patient outcomes | *And you've been through the eye test and the other machine that's looked at the back of you. You then go back into the waiting room, and then the doctor that's from there at the time you go into her room, and she decide, well, it was a lady last time I went. She decided to look at the results of this eye test that I’ve just had, and she'll say right, there's no buildup of fluid in the back of your eye this time. It's fine. You don't need an injection or she'll say the fluid is building up in the back of your eye. It's not looking good. I think you'd be better with the injection this time which has usually happened for quite a long time when I went. But the last two times I’ve been, she said the news is that the fluid at the back of the eye is not there. It's not building up, so we don't have to inject. And I thought, oh well, thank you very much. Yeah, and she'll tell you she'll look at the notes that have gone through, and she'll tell you what... what happened. And after that, she'll say, right, you can go. So off I went. And she'll say right... she calls the nurse saying, and she’d say right, we'll get you ready for the injection, and she does the injection there and then. (patient, site 3)* |
| Mobility issues | *I have somebody that helps me and she has a car so she takes me. If not, I get a taxi, and it’s extremely easy as opposed to the [name of hospital] which is absolutely unbelievable and difficult to get to (patient receiving care in the community, site 3)* |
| Physical environment | *The… the only other sort of slight issue that we have at [opticians] in [town] is that the OCT machine is upstairs and there’s nothing available downstairs. So, the… the research team at York Hospital are aware of that. So that they are… they are selecting patients that… that, you know, are mobile and are able to make it upstairs for the… for the OCT scans. But in the long… in the longer term, you know, that might present some issues, possibly not during the… you know, the study period. But, you know, afterwards if people were to come into community care in the longer term, there’d have to be some arrangements for having an OCT downstairs to enable access (healthcare professional, site 4)* |
| Patient understanding of condition | *They are monitoring my left eye at the moment because I have some signs of what I call break out, but it’s outside my main field of vision and it has actually been there for many years (patient, site 4)* |
| Other patient sight or health issues | *That’s what she said. It's just the start of them that we've picked up on the last two times when with the machine that looks at the eye. We’ve picked up the start of two of a very small... two, I think she said cataract at the back of your good eye and one in the bad eye. Well I’m not too bothered about the bad eye because I can't see out of that anyway, but this good eye, so I mean the cataracts grow fast. She said some do; some don’t. It just depends but now, we know that they're just starting; we will keep an eye on those. But she said don't worry, don't worry. When it comes to time, we can do something about it because I came out feeling thinking of dealing... this is the end sort of thing. But no, no, no. We can treat cataracts. We can treat them now, so don't worry about that. If the worse go to the worse, we can take them off, you know, when they're getting a bit bigger. (patient site 4)* |
| Transport and location | *‘Oh yeah, it makes it… makes me a lot better because my wife can drive me and then coming out, she can join me home. So the only bad thing is you can go shopping.’ (patient site 2)* |
| FENETRE training | *It improved my knowledge of things, obviously. You know, OCT is not something that we’re sort of specifically trained in … for me, it was a challenge to sort of improve my skills, improve my knowledge, and from that perspective I think, you know, it did help in that respect.* *(community optometrist site 1)* |
| Alternate pathway suggestions | *I do wonder when speaking to the patients how they are demarcating their eye conditions and how much knowledge they have of what the outcomes are and what might come next. It doesn’t always feel that transparent. (healthcare professional, site 3)* |
| Purpose of FENETRE | *It's... it's well organised. I get good communication. They treat me with the... various people who contact me with the utmost respect. Not deference which isn't required, I wouldn't want. But they are respectful and careful in what they say and that's... that's great. That's important. I don't have any reds under the beds problem, so I don't see this study as a trojan horse to get my care downgraded. Other people might do that, and I don't feel like that. (healthcare professional, site 4)* |
| Patient referral or history | *I first noticed a distortion in my vision when I was on holiday in Ibiza, and I thought it was something to do with the sun. I came back, when I came back, I made an appointment to see my doctor, unfortunately so that was another few weeks, and she said, “you must go to an optician”. I went to the opticians, and fortunately it was a young lady who just started, and she had been trained at Moorfields, and she said to me what it was, and told me that I had to get to an A&E department that day, and gave me a few options. And [the hospital] is the easiest one for me to get to. It’s not easy to get to the other ones that are in my region. And that was it, we went from there. I can’t remember how many years ago, it must be about seven, eight. (patient, site 1)* |
| Covid-19 | *So a patient mentioned that they were quite pleased they didn’t have to go into the hospital because there’s a whole team that see her at the hospital, so she felt safer coming straight into the room and it’s just me taking her through the whole process. So she feels safer with that. (healthcare professional, site 3)* |
| Cost of care | *Well, I… I think realistically, if you look at the remuneration, it… it probably doesn’t even cover the… the chair time as it stands. So, there’s… there’s two ways that opticians could look at it. One is that it’s… if they didn’t have a full appointment diary, then it’s… you know, it’s extra income. But assuming that they did have a full appointment diary, the only advantage that they get out of it is… is to have a retained patient in their… in their practice. But, in actual terms… you know, in terms of profit and, you know, business profitability and so on, the remuneration at the moment simply doesn’t cover the cost of providing the service. So, I think probably, you know, optometrists are being… they’ve been happy to take part in the study, perhaps out of interest and so on. (healthcare professional, site 4)* |
