# Supplementary material for: Comparing community-based monitoring to hospital-based care of patients with quiescent age-related macular degeneration: a qualitative study of patient and practitioner perspectives on acceptability and access
Source: BMJ Open. 2026 Feb 4;16(2):e101379. doi: 10.1136/bmjopen-2025-101379 (PMC12878339; doi:10.1136/bmjopen-2025-101379)
Supplement: Supplementary file 2 [file bmjopen-2025-101379.reviewer_comments.pdf]

PEER REVIEW HISTORY

BMJ Open publishes all reviews undertaken for accepted manuscripts. Reviewers are asked to complete a checklist review form and are provided with free text boxes to elaborate on their assessment. These free text comments are reproduced below.

ARTICLE DETAILS

Title (Provisional)

Comparing community-based monitoring to hospital-based care of patients with quiescent age-related macular degeneration. A qualitative study of patient and practitioner perspectives on acceptability and access

Authors

Vougioukalou, Sofia; Read, Simon M; Csontos, Judit Katalin; Jones, Aled; Jaber, Alijazy; Sharma, Anitta; Balaskas, Konstantinos

---

VERSION 1 - REVIEW

**Reviewer**	**1**
**Name**	**Pazo, Emmanuel Eric**
**Affiliation**	**Tianjin Medical University Eye Hospital, Ophthalmology**
**Date**	**24-Jun-2025**
**COI**	**None**

This paper used qualitative process evaluation to explore the acceptability of community-based monitoring versus hospital-based care for patients with quiescent neovascular age-related macular degeneration (QnAMD). Conducted as part of the FENETRE randomized controlled trial, the study involved interviews with 25 patients and 16 healthcare professionals across six hospitals and six community practices. Patients generally expressed high satisfaction with community care, highlighting convenience, familiarity, and good communication with staff. Professionals supported the model but raised concerns about data sharing limitations, accessibility of some community clinics, and financial sustainability post-trial. Despite some patient preference for hospital settings, community-based follow-up was broadly accepted as safe and feasible. Key determinants of acceptability included proximity of the clinic, ease of transport, and continuity with familiar providers. The study concludes that community-led QnAMD care is acceptable and potentially scalable, though future research should address infrastructure, equity, and economic viability for wider implementation.

This manuscript offers valuable insights into the acceptability of community-based care for QnAMD. It is thorough and well-organized, with rich qualitative data. Some revisions in

clarity, formatting, and conciseness are needed, particularly in the Results and Discussion sections. Enhancing synthesis and tightening the prose will significantly improve readability and impact.

Comment -1. Regarding the title on Page 2, Lines 1 to 2: The title is clear and descriptive. However, consider rephrasing “Is community-based monitoring inferior…” to a more neutral framing like “Comparing community-based and hospital-based monitoring…” to avoid implying a hypothesis of inferiority before presenting results.

Simulationausly, the authors can seek help from equator-network.org, reporting-guidelines/ (https://www.equator-network.org/reporting-guidelines/) in framing an appreciate title.

Comment-2. On Page 2, Lines 15 to 25: The abstract is generally well-written, but the phrase “specific mentions of the professionalism, competence, and communication skills” could be streamlined, perhaps just say “patients highlighted staff professionalism and clear communication.”

Comment-3. Page 3, lines 17 to 22: You mention shared care models in Finland and Australia, this is a strong contextual anchor. However, it might help to briefly state whether these international models were community or hospital-driven to match your comparison.

Comment-4. Page 3, Line 39 “About two-thirds of departments…” Kindly consider clarifying whether this refers specifically to UK departments or a broader international sample.

Comment 5. Page 5, Line 6: “Sampling of sites was performed purposively…” perhaps add a sentence explaining why purposive sampling was the most suitable approach for this evaluation.

Comment 6: page 6, Line 29: The line “no previous relationship existed between researchers and participants” helps understand bias risk. It would be useful to briefly mention any steps taken to ensure reflexivity.

Comment 7: Page 7, Lines 3, 4: It says 21 themes were identified, but only a few are discussed in the main text. Consider briefly summarizing the others or clarifying that only representative examples are shown.

Comment 8: page 8, Lines 1 to 25: The direct quotes from patients and professionals are rich and illustrative. However, the volume of quotes may overshadow synthesis. Consider balancing thematic synthesis with direct excerpts.

Comment 9: page 10. line 33: Accessibility concerns are repeated twice (“Reportage of this issue…”) almost word-for-word. This looks like a copy-paste error and should be corrected for clarity.

Comment 10: Page 13, Line 1: The summary is effective. Still, it may help to explicitly state that “community monitoring is not inferior” if that aligns with the trial’s clinical findings (this seems implied but not clearly stated).

Comment 11: page 13, Lines 18 to 22: When discussing mobility issues and physical access, you could strengthen this by referencing specific accessibility regulations or guidelines (e.g., Equality Act 2010).

Comment 12: page 14, Lines 46 to 48: The limitation regarding the small number of hospital staff interviewed (only one ophthalmologist) is significant. This should perhaps be emphasized further in the discussion, especially since acceptability from the hospital side is central to implementation.

Comment 13- Page 16, Author Contributions: AJ appears twice with identical initials, once for supervision and once for data collection. Please check if these refer to the same author or if this is a formatting error.

Comment 14: Some sections are overly wordy and could benefit from more concise summaries. Consider reducing redundancy to improve flow (e.g., repetition in training and data transfer discussions).

Comment 15: pge 16, line 42-Ethics approval is clearly stated and appropriate. However, under "Data Sharing Statement," the explanation about data being unavailable could more explicitly state what procedures exist for data requests (e.g., contacting the corresponding author).

Comment 16: Figure 1 should include a study design flowchart to provide the readers a detailed view of this interesting research following equator-network.org, reporting-guidelines/ (https://www.equator-network.org/reporting-guidelines/)

**Reviewer**	**2**
**Name**	**Lin, Jianqiang**
**Affiliation**	**Sun Yat-Sen University Zhongshan Ophthalmic Center**
**Date**	**03-Jul-2025**
**COI**	**None**

This manuscript presents a well-conducted qualitative process evaluation embedded within the FENETRE randomized controlled trial, examining the acceptability of community-based follow-up for patients with quiescent QnAMD. The research question is timely and highly relevant, considering the growing pressure on hospital eye services and the interest in decentralizing chronic disease management.

The authors use a theory-informed qualitative approach and incorporate perspectives from both patients and healthcare professionals across multiple sites. The thematic analysis is thoughtful, and the conclusions are generally supported by the data. However, several areas

could benefit from clarification or deeper discussion to strengthen the paper's contribution and interpretative clarity.

1.Only 9 patients from the hospital group were interviewed, and some were unaware of the existence of a community care arm. This informational asymmetry may affect the balance and comparability of perspectives. Please elaborate on how this was considered during analysis and interpretation.

2.While the conclusion states that community-based care was "broadly acceptable," a number of participants expressed clear preferences for hospital follow-up. Could the authors clarify the distinction between passive acceptability and active patient preference, especially in light of future implementation strategies?

3.Multiple community professionals reported difficulty with incomplete OCT image data due to IT incompatibility. It would be helpful if the authors could quantify how often such issues impacted decision-making or led to additional referrals. This would strengthen the understanding of operational feasibility.

4.While the manuscript discusses community practitioners' concerns over inadequate remuneration and workflow burden, the analysis remains anecdotal. Have the authors considered conducting a preliminary cost-effectiveness or budget impact estimation (e.g., chair time vs. NHS reimbursement)? Such data could help assess the long-term viability of the model, especially in corporate chain practices.

5.Although the framework is cited, the mapping between themes and theoretical constructs (e.g., affective attitude, burden, intervention coherence) is not always explicit. A summary table aligning thematic findings with theoretical components would enhance clarity and rigor.

VERSION 1 - AUTHOR RESPONSE

Reviewer: 1

1.1. Regarding the title on Page 2, Lines 1 to 2: The title is clear and descriptive. However, consider rephrasing "Is community-based monitoring inferior..." to a more neutral framing like "Comparing community-based and hospital-based monitoring..." to avoid implying a hypothesis of inferiority before presenting results.

7th of August 2025

Simulationausly, the authors can seek help from equator-network.org, reporting- guidelines/ (https://www.equator-network.org/reporting-guidelines/) in framing an appreciate title.

>> Title updated

1.2. On Page 2, Lines 15 to 25: The abstract is generally well-written, but the phrase "specific mentions of the professionalism, competence, and communication skills" could be

streamlined, perhaps just say “patients highlighted staff professionalism and clear communication.”

>> Sentence clarified

1.3. Page 3, lines 17 to 22: You mention shared care models in Finland and Australia, this is a strong contextual anchor. However, it might help to briefly statewhethertheseinternationalmodelswerecommunity orhospital-drivento match your comparison.

>> Further explanation with details of these shared eye care models added. The authors mentioned that the arrangements between independent optometrists and ophthalmologists were informal as funding came from a mix of patient out- of-pocket fees and government reimbursements.

1.4.Page3,Line39“Abouttwo-thirdsofdepartments...”Kindly consider clarifying whether this refers specifically to UK departments or a broader international sample.

>> Specific mention to the UK added

1.5. Page 5, Line 6: “Sampling of sites was performed purposively...” perhaps add a sentence explaining why purposive sampling was the most suitable approach for this evaluation.

>> We included information from the protocol which stated that: ‘The sample will not be stratified per se; instead a purposive maximum variation sample will be selected to generate a broad range of views on whether and how the clinic is acceptable to participants.’

1.6: page 6, Line 29: The line “no previous relationship existed between researchers and participants” helps understand bias risk. It would be useful to briefly mention any steps taken to ensure reflexivity.

>> We changed the position of the statement regarding the relationship between researchers and participants to enhance clarity. We also added that notes were taken during the interviews and key observations were shared between researchers during regular meetings.

1.7: Page 7, Lines 3, 4: It says 21 themes were identified, but only a few are discussed in the main text. Consider briefly summarizing the others or clarifying that only representative examples are shown.

>> We have clarified the wording to explain in more detail the data presented here which covers all 21 themes. They were organised in 5 broad categories.(Acceptability of community care monitoring, The physical environment, The organisation of care, Issues specific to patient experience, and Issues specific to staff experience). Full details are presented in Supplementary table 6. In this paper we have aggregated the most pertinent data from

across those 21 themes into the 3 sections as most quotes were attributed to a range of themes as they often overlapped.

1.8: page 8, Lines 1 to 25: The direct quotes from patients and professionals are rich and illustrative. However, the volume of quotes may overshadow synthesis. Consider balancing thematic synthesis with direct excerpts.

>> We have kept only one illustrative quote in the main body of the text and move additional quotes in Supplementary file 7.

1.9: page 10. line 33: Accessibility concerns are repeated twice ("Reportage of this issue...") almost word-for-word. This looks like a copy-paste error and should be corrected for clarity.

>> The duplicate has been removed

1.10: Page 13, Line 1: The summary is effective. Still, it may help to explicitly state that "community monitoring is not inferior" if that aligns with the trial's clinical findings (this seems implied but not clearly stated).

>> We have added that this complements the main trial findings that showed that community monitoring is not inferior to hospital monitoring referring to the non peer-reviewed preprint in the Lancet
https://papers.ssrn.com/sol3/papers.cfm?abstract_id=5278556

1.11: page 13, Lines 18 to 22: When discussing mobility issues and physical access, you could strengthen this by referencing specific accessibility regulations or guidelines (e.g., Equality Act 2010).

>> We have added that issues around accessibility, particularly with regard to older people, may require further investigation so as to comply fully with the Equality Act 2010.

1.12: page 14, Lines 46 to 48: The limitation regarding the small number of hospital staff interviewed (only one ophthalmologist) is significant. This should

perhaps be emphasized further in the discussion, especially since acceptability from the hospital side is central to implementation.

>> We explicitly mention in the Limitations that there was limited recruitment of hospital professionals meaning that interpreting any issues around perceptions of community optometry in that setting is difficult and requires further exploration in future studies.

1.13- Page 16, Author Contributions: AJ appears twice with identical initials, once for supervision and once for data collection. Please check if these refer to the same author or if this is a formatting error.

>> Added differentiation in the acronym to make it easier to identify the two co- authors

1.14: Some sections are overly wordy and could benefit from more concise summaries. Consider reducing redundancy to improve flow (e.g., repetition in training and data transfer discussions).

>> We have edited the section on training and data transfer to improve flow.

1.15: pge 16, line 42-Ethics approval is clearly stated and appropriate. However, under "Data Sharing Statement," the explanation about data being unavailable could more explicitly state what procedures exist for data requests (e.g., contacting the corresponding author).

>> Participants did not consent to data sharing and therefore raw data cannot be shared due to privacy and ethical restrictions. All data requests should be submitted to the corresponding author for consideration

1.16: Figure 1 should include a study design flowchart to provide the readers a detailed view of this interesting research following equator-network.org, reporting-guidelines/ (https://www.equator-network.org/reporting-guidelines/) >> The Flowchart for the clinical trial is included in the protocol paper (see figure pasted below which is figure 1 in https://bmjopen.bmj.com/content/11/5/e049411). The process evaluation reported in this article was conducted independently from the main trial. We followed the COREQ guidelines for reporting the process evaluation. If the reviewer would like us to use a different specific flow diagram then please specify the exact reporting guidelines.

Reviewer: 2

2.1. Only 9 patients from the hospital group were interviewed, and some were unaware of the existence of a community care arm. This informational asymmetry may affect the balance and comparability of perspectives. Please elaborate on how this was considered during analysis and interpretation.

>> We have clarified that they were all aware that they could have gone to the community. This was not a blinded (masked) trial. As per protocol the only masking in this study will be the statisticians and health economists so that the analyses can be performed masked to treatment. Whilst participants and clinicians cannot be masked to management status, those conducting the analysis will be masked.

2.2.While the conclusion states that community-based care was "broadly acceptable," a number of participants expressed clear preferences for hospital follow-up. Could the authors clarify the distinction between passive acceptability and active patient preference, especially in light of future implementation strategies?

>> This has been addressed in the discussion

2.3.Multiple community professionals reported difficulty with incomplete OCT

image data due to IT incompatibility. It would be helpful if the authors could quantify how often such issues impacted decision-making or led to additional referrals. This would strengthen the understanding of operational feasibility. >> This wasn't within the study remit of the qualitative arm, but might help to explain other trends in the wider study data, e.g., referrals back into hospital care.

2.4. While the manuscript discusses community practitioners' concerns over inadequate remuneration and workflow burden, the analysis remains anecdotal. Have the authors considered conducting a preliminary cost-effectiveness or budget impact estimation (e.g., chair time vs. NHS reimbursement)? Such data could help assess the long-term viability of the model, especially in corporate chain practices.

>> A health economic evaluation will be reported separately. It includes a within trial analysis of Costs and Contributions, where the cost of the intervention itself will be subject to a micro-costing exercise, which include staff, equipment, administration and any other relevant costs for delivering the intervention. The costs of participant time and travel when accessing care will be informed by the results of a bespoke time and travel questionnaire. It also includes:

1) Principal economic outcome: Incremental cost per quality adjusted life year (QALY) gained over the estimated patient lifetime estimated from an economic model informed by trial data.

2) Use of health services and patient costs collected via eCRF and participant completed questionnaires

3) Costs of interventions and subsequent care to the NHS modelled over the estimated lifetime

4) Budget impact to the NHS

5) Modelled estimates of visual impairment and QALYs based on responses to the EQ-5D-5L

2.5. Although the framework is cited, the mapping between themes and theoretical constructs (e.g., affective attitude, burden, intervention coherence) is not always explicit. A summary table aligning thematic findings with theoretical components would enhance clarity and rigor.

>> We have provided a table which includes analysis that we decided in the end not to include because we felt it did not add a new dimension to our understanding of intervention acceptability, mainly because when the interviews were conducted without TOA in mind so applying TOA to the data felt like a retrofit. However, if the reviewer feels that it add rigour then we are very happy to

include as we wanted the analysis to have a theoretical component.

VERSION 2 - REVIEW

**Reviewer**	**1**
**Name**	**Pazo, Emmanuel Eric**
**Affiliation**	**Tianjin Medical University Eye Hospital, Ophthalmology**
**Date**	**22-Sep-2025**
**COI**	

“Comparing community-based monitoring to hospital-based care of patients with quiescent age-related macular degeneration. A qualitative study of patient and practitioner perspectives on acceptability and access”; this qualitative process evaluation, nested within the FENETRE randomized trial, examines the acceptability of community-based monitoring versus hospital follow-up for quiescent neovascular AMD (QnAMD). Interviews with 25 patients and 16 clinicians across six hospitals and six community optometry practices explored experiences, logistics, and resourcing using a Theory of Acceptability lens. Patients generally viewed community clinics positively, citing professional staff and clear communication; proximity to home and transport convenience strongly influenced acceptability. Some hospital-randomized patients still preferred hospital care. Clinicians felt capable of delivering community QnAMD services but highlighted structural barriers: incompatible IT systems for OCT image and video transfer, hidden administrative costs threatening financial sustainability, and building accessibility issues at some practices. The authors conclude that community follow-up by trained non-medical practitioners is broadly acceptable, while calling for evaluation in routine practice and assessment of long-term financial viability. Trial registration NCT03893474.

This is a timely, well-designed qualitative companion to a large RCT. The manuscript would benefit from sharper reporting of sampling, analytic procedures, and a clearer bridge to implementation, costs, and equity.

Comments

1. Figure 1 of the manuscript should include a detailed Equator-network study design flowchart (https://www.equator-network.org/). Altman, Douglas G., et al. "EQUATOR: reporting guidelines for health research." The Lancet 371.9619 (2008): 1149-1150.

2. On pg 4, Lines 91-99, 97-99: Positioning relative to the parent RCT: You state the RCT enrolled 704 participants and tested non-inferiority and budget impact to the NHS. Please summarize the key RCT outcomes here to contextualize acceptability findings and note whether non-inferiority was met.

3. Page 4, Lines 103-118: Sampling clarity and transferability: Provide a table of participant characteristics (age, gender, socioeconomic status, rural vs urban) to help readers judge transferability of views; currently the site sampling is well described, but participant descriptors are sparse.

4. Page 2, Lines 27-31; Methods section header. Analytic framework and rigor: The authors mention using an acceptability framework; please specify how codes mapped to constructs and whether double-coding or consensus procedures were used to enhance trustworthiness. Add a brief codebook description and example quotes per theme.

5. On Page 2, Lines 41-43: Equity and accessibility: Building accessibility problems in community sites warrant deeper analysis. Consider discussing reasonable adjustments and potential inequities for patients with low mobility or low vision, and whether hospital sites were comparatively better or worse.

6. Page 2, Lines 36-40: Digital infrastructure: The IT interoperability barrier for OCT images is central. Please specify the file types, bandwidth, image volumes, and governance hurdles encountered, and outline practical solutions or standards needed for scale-up.

7. Page 2, Lines 40-41; Conclusion section. Economic sustainability: "Hidden costs" are mentioned; quantify typical time, equipment, and admin costs and relate them to reimbursement models. A brief sensitivity analysis or triangulation with the RCT's economic evaluation would strengthen conclusions about sustainability.

8. On page 2, Lines 32-36: Preference heterogeneity: Some hospital-randomized patients preferred hospital follow-up. Clarify whether preference aligned with prior experience, comorbidities, or travel distance, and whether preference changed over time.

9. On page 2, Lines 130-132, COVID-19 disruption: The authors note a 102-day suspension. Discuss how this affected sample composition, recruitment of harder-to-reach patients, and the salience of infection-control benefits of community care.

10. Page 2, Lines 118-125; 107-113; linking themes to implementation: The conclusion asserts safety and acceptability; consider adding a short implementation roadmap addressing data-sharing agreements, training refreshers, audit pathways, and minimum accessibility standards.

Minor comments

11. Abstract wording: Consider tightening the Results to foreground the three most policy-relevant findings: proximity, IT interoperability, and costs. Page 2, Lines 32-43.

12. Title and design labelling: The title now reflects a qualitative study; ensure consistent phrasing across the manuscript. Page 2 title; Page 34 editorial note.

13. Strengths and limitations: "Data saturation was reached" would benefit from explicit criteria and an audit trail description. Page 2, Lines 127-129.

14. The cited literature is appropriate and includes global AMD burden, shared-care precedents, the FENETRE protocol, and qualitative methods/acceptability frameworks. Consider updating the parent FENETRE RCT results citation if a peer-reviewed version supersedes the "Preprints with the Lancet, 2025" entry, and add a standards reference for health IT interoperability if discussing OCT data transfer.

**Reviewer**	**2**
**Name**	**Lin, Jianqiang**
**Affiliation**	**Sun Yat-Sen University Zhongshan Ophthalmic Center**
**Date**	**06-Sep-2025**
**COI**	

The authors have addressed the previous reviewer comments carefully. The revisions make the manuscript clearer and more reasonable in methodology. The quality of the paper has been improved. At present, the study is presented in a clear and acceptable way. I recommend the manuscript for publication in BMJ Open.

VERSION 2 - AUTHOR RESPONSE

Response to reviewer 2
1.Figure 1 of the manuscript should include a detailed Equator-network study design flowchart (https://www.equator-network.org/). Altman, Douglas G., et al. "EQUATOR: reporting guidelines for health research." The Lancet 371.9619 (2008): 1149-1150.
>> The Equator-network study design flow chat that relates to the main trial can be found in the Lancet Preprints of the main trial findings (Sharma et al. 2025, page 10). We used the COREQ reporting framework for qualitative research (Supplementary file 5).

2. On pg 4, Lines 91-99, 97-99: Positioning relative to the parent RCT: You state the RCT enrolled 704 participants and tested non-inferiority and budget impact to the NHS. Please summarize the key RCT outcomes here to contextualize acceptability findings and note whether non-inferiority was met.
>> The process evaluation was conducted independently from the parent trial. We have included a discussion of the main trial findings as per Sharma et al. 2025 (Lancet pre-print) in the Discussion section. For the reviewers' reference these were the key findings:
Findings: Of 704 recruited participants between October 8, 2019, and January 31, 2023, 635 (90.2%) completed follow-up (348 hospital, 287 community). Community optometry missed fewer reactivations (11/287 vs 27/348; difference −3.9%, 95% CI −7.4 to −0.3), meeting non-inferiority (adjusted OR 0.51, 95% CI 0.24–1.07; p=0.08). Community optometry generated higher false-positive referrals (24/287 vs 12/348; difference 4.9%, 95% CI 0.9–9.0; p=0.01).
Interpretation: Community-based nAMD monitoring by trained optometrists is safe for detecting nAMD disease activity, with a small increase in unnecessary hospital referrals. The study's methodological approach may offer a transferable framework for evidence-generation in shared-care clinical models, easing hospital demand and improving access without compromising safety.

3. Page 4, Lines 103-118: Sampling clarity and transferability: Provide a table of participant characteristics (age, gender, socioeconomic status, rural vs urban) to help readers judge transferability of views; currently the site sampling is well described, but participant descriptors are sparse.

>> We do not want to report specific demographic data to avoid identification. Particularly as some of these participants are still under hospital care. We can however report that all patient participants were 55 or older as that was in the RCT inclusion criteria and participants were retired. All sites apart from site 6 were urban.

4. Page 2, Lines 27-31; Methods section header. Analytic framework and rigor: The authors mention using an acceptability framework; please specify how codes mapped to constructs and whether double-coding or consensus procedures were used to enhance trustworthiness. Add a brief codebook description and example quotes per theme.
>> Consensus was reached through a series of meetings between researchers SR and AJ to discuss structure and content. JC used these codes to analyse the consequent interviews. Further analysis was undertaken by SV in consultation with SR and JC and four bucket themes and the way they map to the Theory of Acceptability framework was applied to the data. In the Supplementary files we have presented the codebook and how it is linked to the Theory of Acceptability framework (file 6). File 7 provides example quotes for the Acceptability theme. We have added an additional file (8) with quotes for the other key themes.

5. On Page 2, Lines 41-43: Equity and accessibility: Building accessibility problems in community sites warrant deeper analysis. Consider discussing reasonable adjustments and potential inequities for patients with low mobility or low vision, and whether hospital sites were comparatively better or worse. Thank you for this feedback. We have embellished the reference to this issue in the Abstract and Discussion to include issues prevalent to accessing some hospital sites and community practices.

6. Page 2, Lines 36-40: Digital infrastructure: The IT interoperability barrier for OCT images is central. Please specify the file types, bandwidth, image volumes, and governance hurdles encountered, and outline practical solutions or standards needed for scale-up.
>> Thank you for bringing this up. We did not collect data at this technical level as the aim of the process evaluation was to identify barriers and facilitators broadly. We added technical information about IT systems in the Data transfer and technological infrastructure section and also directed the reader to the trial protocol (Learoyd et al. 2021) published in the BMJ Open.

7. Page 2, Lines 40-41; Conclusion section. Economic sustainability: "Hidden costs" are mentioned; quantify typical time, equipment, and admin costs and relate them to reimbursement models. A brief sensitivity analysis or triangulation with the RCT's economic evaluation would strengthen conclusions about sustainability.

>> The analysis in this specific paper has been conducted independently of the main trial as we did not want to influence the analysis that different teams have made. We have referred to the findings of the parent trial, i.e., Sharma et al, 2005, where these considerations are covered in more depth. For this article, we have also removed the word 'hidden' because it had potential to be misunderstood. We were referring to costs that would be encountered should this intervention be implemented in real-world settings outside of the clinical trial context.

8. On page 2, Lines 32-36: Preference heterogeneity: Some hospital-randomized patients preferred hospital follow-up. Clarify whether preference aligned with prior experience, comorbidities, or travel distance, and whether preference changed over time.
>> We did not collect data over time. Preference was aligned with ease of access as discussed. We have clarified in the abstract that preference heterogeneity was mainly mediated by prior experience of hospital care and travel distance.

9. On page 2, Lines 130-132, COVID-19 disruption: The authors note a 102-day suspension. Discuss how this affected sample composition, recruitment of harder-to-reach patients, and the salience of infection-control benefits of community care.

>> Recruitment was undertaken by the research nurses in the Hospital Eye Service teams who conducted qAMD follow ups. The research team was not responsible for recruitment. The impact on

recruitment to the trial is reported by Sharma et al. 2025.

10. Page 2, Lines 118-125; 107-113; linking themes to implementation: The conclusion asserts safety and acceptability; consider adding a short implementation roadmap addressing data-sharing agreements, training refreshers, audit pathways, and minimum accessibility standards.

Assessing implementation feasibility was beyond the remit of the process evaluation. Further research would be needed to optimise the uptake of community monitoring by diverse community optometry practices that serve different populations. The main trial explored this topic and reported the following (Sharma et al. 2025: 16):

'A programme of applied health research would generate evidence evidence to guide the implementation of integrated care for community monitoring of nAMD. This could include the integration of digital technologies, such as automated notifications, multi-channel communications, and remote specialist advice through teleophthalmology platforms to optimise integrated care and maximise patient retention in community care.'

11. Abstract wording: Consider tightening the Results to foreground the three most policy-relevant findings: proximity, IT interoperability, and costs. Page 2, Lines 32-43.
>> We have reworded the Results section of the abstract to raise the prominence of these three issues.

12. Title and design labelling: The title now reflects a qualitative study; ensure consistent phrasing across the manuscript. Page 2 title; Page 34 editorial note.
>> We have updated the wording to ensure consistent phrasing throughout the text and supplementary files.

13. Strengths and limitations: "Data saturation was reached" would benefit from explicit criteria and an audit trail description. Page 2, Lines 127-129.
>> Our definition of data saturation refers to the state in the analysis where no new information, themes, or insights emerge from the data in relation to the acceptability of the trial. This was achieved through discussions between the researchers during the data analysis phase as well as during the period when the paper was being developed.

14. The cited literature is appropriate and includes global AMD burden, shared-care precedents, the FENETRE protocol, and qualitative methods/acceptability frameworks. Consider updating the parent FENETRE RCT results citation if a peer-reviewed version supersedes the "Preprints with the Lancet, 2025" entry, and add a standards reference for health IT interoperability if discussing OCT data transfer.

>> We do not have a peer-reviewed version of the Preprints. The data transfer system used for FENETRE was developed in-house at Moorfields by the R&D IT team. In the study design we say the following regarding Information security standards for the data transfer system/electronic database: Electronic Database: The front end will use a bespoke Microsoft Visual Studio application and the back end (data storage) will be hosted on Moorfields Research Database SQL servers. Local hospital trial-coordinator and local optometry staff will manage uploading both image and patient data (case report forms) on a monthly basis on a bespoke eCRF. This will be done using Moorfields remote log-in tokens, where the user will log onto clinical services and see the local drives of the PC they are using. The database will be validated to GAMP 5 standards.

This information has been included in the manuscript.

VERSION 3 - REVIEW

Reviewer 1

Name Pazo, Emmanuel Eric

Affiliation Tianjin Medical University Eye Hospital, Ophthalmology

Date 17-Nov-2025

COI

This qualitative process evaluation explores the acceptability and access implications of community-based monitoring for quiescent neovascular AMD (QnAMD) within the FENETRE program. The manuscript states that the parent RCT enrolled 704 participants and tested non-inferiority and budget impact, while this study interviewed 41 stakeholders to understand acceptability and access (patients, optometrists, ophthalmologists). The work addresses a clinically important service-delivery question. Methods and analysis are largely appropriate for a qualitative study that uses framework analysis aligned to the Theory of Acceptability. Key improvements are needed in reporting demographics, sampling transparency, and clearer integration with the parent trial's main outcomes to help readers judge transferability and policy relevance. Overall, the study is valuable and publishable with minor revision, especially after strengthening COREQ-aligned reporting and tightening language for clarity (see detailed, page-line comments and edits).

1. Regarding contextualizing findings with parent RCT outcomes: p.41, lines 13-30: The authors describe the RCT and say further information is in Lancet Preprints. Add one or two sentences summarizing whether non-inferiority was met and briefly note the false-positive trade-off, so readers can connect acceptability themes to safety and utilization.

2. Specify analytic rigor and codebook handling: p.41, comment block and responses; p.61, lines 248-249 and 255-260: You note use of framework analysis and the Theory of Acceptability. In the main text, briefly explain double-coding or consensus procedures, codebook development, and how codes are mapped to constructs. State who coded what, how disagreements were resolved, and how the authors achieved data saturation or stopping criteria.

3. Report interview logistics consistently: p. 24, lines 161-170 and p. 62, lines 167-171. Data collection over the phone versus face-to-face across the pandemic requires one clear sentence in Methods with timing, rationale, and any reflexivity on how the mode may have influenced narratives.

4. Make acceptability dimensions explicit and linked to data: p.37, lines 13-27: You indicate major and minor themes are clear. In Results, signpost each theme with a subheading that corresponds to the Theory of Acceptability dimensions, for example affective attitude, burden, ethicality, opportunity cost, perceived effectiveness, intervention coherence, self-efficacy. Add one representative quote per theme in the main text and move additional quotes to a supplementary table.

5. Economic sustainability claims need sourcing or softer language: p.73, lines 1-12 and p.70, lines 1-10: You revised "hidden costs," which is good. In the Discussion, either reference the parent trial's economic analysis when you comment on sustainability or soften language to "participants perceived staffing, equipment, and admin costs as barriers, which aligns with concerns raised by practitioners." Include a short note that a full cost analysis sits within the main trial papers.

6. Close the loop with implementation pointers: The response notes implementation is beyond scope, which is fair. Still, a short paragraph that translates your themes into practical next steps will help. For example: interoperable IT agreements, periodic refresher training, audit and feedback, and minimum accessibility standards for practices. Present this as implications rather than a prescriptive roadmap.

7. Limitations: Add a sentence acknowledging that not tracking refusals limits assessment of selection bias. Then note that telephone interviews may under-capture nonverbal cues.

8. "This study builds on the pilot and aims to assess the acceptability of community follow-up for QnAMD led by suitably trained non-medical practitioners." Suggestion: "This study builds on prior pilot work. We assess how acceptable community follow-up is for QnAMD when led by trained non-medical practitioners."

9. "We have removed the word 'hidden' because it had potential to be misunderstood." Suggestion: "We removed the term 'hidden,' since readers could misinterpret it."

10. "Preference heterogeneity was mainly mediated by prior experience of hospital care and travel distance." Suggestion: "Preferences differed. Prior hospital experience and travel distance were the main drivers."

VERSION 3 - AUTHOR RESPONSE

1. Regarding contextualizing findings with parent RCT outcomes: p.41, lines 13-30: The authors describe the RCT and say further information is in Lancet Preprints. Add one or two sentences summarizing whether non-inferiority was met and briefly note the false-positive trade-off, so readers can connect acceptability themes to safety and utilization.

>> Thank you for this comment, we appreciate that readers do not want to go back and forth between papers but we are cautious not to report unpublished data extensively. We have added the following sentence summarising the trial findings: ‘Community-based nAMD monitoring by trained optometrists is safe for detecting nAMD disease activity, with a small increase in unnecessary hospital referrals. Community monitoring achieved very high sensitivity (close to hospital-based care), meaning almost all true recurrences were detected. However, this came at the cost of lower specificity, leading to more false

positives—patients flagged for suspected recurrence who did not actually need treatment.'

2. Specify analytic rigor and codebook handling: p.41, comment block and responses; p.61, lines 248-249 and 255-260: You note use of framework analysis and the Theory of Acceptability. In the main text, briefly explain double-coding or consensus procedures, codebook development, and how codes are mapped to constructs. State who coded what, how disagreements were resolved, and how the authors achieved data saturation or stopping criteria.

>> We think this comment refers to the data analysis section on p.43 – 30 to p.43 - . Thank you for this comment, we have added the following sentence to describe the processes we had in place to ensure inter-rater reliability: All researchers (SR, JC and SV) independently coded the transcripts using the framework developed by SR for the FENETRE pilot and double-checked the coding with Professors Aled Jones, Heather Waterman and Molly Courtenay (the last two colleagues retired during the study). SV mapped the codes to Theory of Acceptability constructs and identified illustrative quotes per theme for this paper in discussion with the other researchers. As the data did not match directly all seven aspects of the Theory of Acceptability, the authors decided to structure the paper around the different factors affecting acceptability for each participant group as the arguments were better supported when triangulated with quotes from different participant groups. It is for this reason that the Theory of Acceptability does not feature in the abstract of the main structure of the Results section. The proposed plan was reviewed by and agreed with parent trial leads (KB and AS).

3. Report interview logistics consistently: p. 24, lines 161-170 and p. 62, lines 167-171. Data collection over the phone versus face-to-face across the pandemic requires one clear sentence in Methods with timing, rationale, and any reflexivity on how the mode may have influenced narratives.

>> P. 24 goes up to line 60 and p. 62 goes up to line 60 as well. We have added the following reflections on data collection over the phone at section at the bottom of the Data Collection sub-section: 'Telephone interviews were conducted as a response to restrictions placed by the pandemic, but this was felt to be less of a risk than not collecting their data. Telephone interviews were not felt to have made a big difference in terms of data collection though a possible downside of telephone interviews is that the researchers cannot see non-verbal cues, such as body language and facial expression. On the other hand a positive of telephone interviews is the convenience to the participants which potentially positively influenced recruitment numbers'.

4. Make acceptability dimensions explicit and linked to data: p.37, lines 13-27: You

indicate major and minor themes are clear. In Results, signpost each theme with a subheading that corresponds to the Theory of Acceptability dimensions, for example affective attitude, burden, ethicality, opportunity cost, perceived effectiveness, intervention coherence, self-efficacy. Add one representative quote per theme in the main text and move additional quotes to a supplementary table.

>> We think this relates to the Results section. As we have stated in the Data Analysis section ‘initially, the Theory of Acceptability framework [15] was a starting point guiding the analysis for this paper, though this evolved as data were collaboratively discussed’. Even though the original plan was to structure the entire paper around the Theory of Acceptability domains, we found that there was not enough data to do so as the interviews were not taken with this theoretical framework in mind. We do feel however, that as it informed the analysis it should be included in the Supplementary files.

5. Economic sustainability claims need sourcing or softer language: p.73, lines 1-12 and p.70, lines 1-10: You revised "hidden costs," which is good. In the Discussion, either reference the parent trial's economic analysis when you comment on sustainability or soften language to "participants perceived staffing, equipment, and admin costs as barriers, which aligns with concerns raised by practitioners." Include a short note that a full cost analysis sits within the main trial papers.

>> We think this relates to the last paragraph of the Discussion (p.53 14-25). We have added a sentence in the end that ‘However, this paper only reflects the qualitative process evaluation findings, the main economic analysis of the parent trial will be published at a later date.’

6. Close the loop with implementation pointers: The response notes implementation is beyond scope, which is fair. Still, a short paragraph that translates your themes into practical next steps will help. For example: interoperable IT agreements, periodic refresher training, audit and feedback, and minimum accessibility standards for practices. Present this as implications rather than a prescriptive roadmap.

>> This is a very good point. We have included in the final Conclusions section that: ‘To address these issues, future studies of shared eye services could consider exploring further the full cost of follow-up care, interoperable IT agreements, periodic refresher training, audit and feedback, and minimum accessibility standards for practices. These topics could be taken into consideration by both academics designing shared eye service trials but also practitioners designing new service pathways.’

7. Limitations: Add a sentence acknowledging that not tracking refusals limits assessment of selection bias. Then note that telephone interviews may under-capture nonverbal cues.

>> Thank you for these suggestions, they have been added to bullets 1 and 3.

8. "This study builds on the pilot and aims to assess the acceptability of community follow-up for QnAMD led by suitably trained non-medical practitioners." Suggestion: "This study builds on prior pilot work. We assess how acceptable community follow-up is for QnAMD when led by trained non-medical practitioners."

>> We have included the reviewer's recommendation and updated the text.

9. "We have removed the word 'hidden' because it had potential to be misunderstood." Suggestion: "We removed the term 'hidden,' since readers could misinterpret it."

>> The word hidden has been removed

10. "Preference heterogeneity was mainly mediated by prior experience of hospital care and travel distance." Suggestion: "Preferences differed. Prior hospital experience and travel distance were the main drivers."

>> We have updated the wording
